# Interactive Effects of Trait Self-Control and Stress Appraisals on Blood Pressure Responses to a Laboratory Stressor

**DOI:** 10.1007/s12529-017-9632-9

**Published:** 2017-02-01

**Authors:** Daryl B. O’Connor, Antonia E. Wilson, Rebecca Lawton

**Affiliations:** School of Psychology, University of Leeds, Leeds, England UK

**Keywords:** Personality, Stress, Conscientiousness, Facets, Longevity

## Abstract

**Purpose:**

Stress may play a role in explaining part of the conscientiousness-longevity relationship. Conscientiousness (C) is associated with the appraisals of stressors and its lower-order facets have been shown to differentially moderate the experience of stress in daily life. This study investigated whether the lower-order facet, self-control (SC), moderated the relationship between stress appraisals and blood pressure responses to a laboratory stressor.

**Methods:**

Ninety participants (selected from the upper and lower quartiles for C scores from a sample of 679 participants) were invited to complete the Maastricht Acute Stress Test (MAST). Systolic blood pressure (SBP) and diastolic blood pressure (DBP) were assessed throughout the stress task. Stress appraisals were assessed at baseline.

**Results:**

Blood pressure responses to the MAST were similar in participants who scored high and low in SC. However, primary appraisals were negatively associated with BP reactivity and recovery in the high SC group but not in the low SC group. Moreover, SC was found to moderate the relationship between primary appraisals and SBP and DBP reactivity values, such that higher primary appraisals were associated with lower BP reactivity in individuals high in SC but not in those low in SC. In addition, lower SBP recovery values were observed in the high SC group compared to their low SC counterparts.

**Conclusions:**

These findings indicate that SC may influence health status by modifying the relationship between perceived demands and blood pressure. Moreover, having a greater stake in stressors may yield health benefits in the longer term for individuals high in SC.

## Introduction

It is well established that conscientiousness (C) influences physical health and longevity [1–4]. Data from the Terman Life Cycle Study have shown that people high in C have a significantly reduced risk of dying in any given year [1]. A growing body of work has focused on exploring potential explanatory mechanisms that may transmit these beneficial effects over the life course. For example, Friedman et al. [5] found that the protective influence of childhood conscientiousness on health status was accounted for, in part, by its impact on health behaviours such as alcohol use and smoking. C has also been found to be associated with better health status [6], greater adherence to medication [4] and lower obesity risk across populations [7]. Nevertheless, evidence now strongly suggests that health behaviours only partially account for the relationship with longevity [5, 8–12]. More recent research, including the current study, has started to focus upon additional mechanisms through which C may convey its beneficial effects. One mechanism through which C may affect health and well-being is through its influence on the stress process [8, 12, 13]. However, understanding the relationship between C, health outcomes and stress has become increasingly complex given that it is now firmly established that C is not a unitary construct, but rather a broad domain best conceptualised as a family of related, but distinct, stable tendencies and characteristics known as facets [14]. In 2005, Roberts, Chernyshenko, Stark and Goldberg [14] found that the lower-order structure of C composed of six facets, namely, industriousness, order, self-control, responsibility, traditionalism and virtue. Moreover, Bogg and Roberts [2], in a meta-analysis of the leading behavioural contributors to mortality, showed that C and its facets were differentially associated with health-related behaviours.

### Conscientiousness and the Stress Process

Using a daily diary design, O’Connor et al. [13] examined whether C had the capacity to moderate the effects of daily stressors on health behaviours. These authors found that high C was associated with more adaptive health behaviours in response to daily stressors. For example, participants with higher levels of order (one of the lower-order facets of C) exercised more on days when they experienced daily stressors. These findings were consistent with a stress buffering hypothesis and suggested that (facets of) C may exert part of its positive influence on health by modifying the effects of daily stressors, such that conscientious individuals respond to stress by engaging in more health-enhancing behaviours. This study also observed a direct effect of C on daily hassles, such that higher levels of the self-discipline (similar to self-control) facet of C were associated with the experience of fewer overall daily hassles. More recently, in a longitudinal study examining the relations between C, perceived stress and perceived physical health, perceived stress was found to partially mediate the association between C and perceived physical health [12]. In addition, changes in C were associated with changes in stress, such that increasing C overtime predicted reductions in perceived stress, which were also associated with changes in perceived health. Although the impact on long-term health remains to be seen, it can be concluded that C may exert protective influences through these direct and moderated pathways but its effects are not straightforward.

### Self-Control and the Stress Process

Recent theorising has suggested that the self-control (SC) facet (of C) may play an important role in the stress process. It has been argued by Hofmann, Friese and Strack [15] that individual differences in SC are associated with one’s ability to consciously exert control over maladaptive habitual response tendencies, and the influence automatic and reflective mental processes have over thoughts, emotions and behaviours. In other words, it is possible that different levels of trait SC are likely to influence information processing, appraisals and behaviours in times of stress. Moreover, new research has focussed on the SC facet in relation to understanding individual differences in stress exposure and reactivity. For example, a daily diary study conducted by Galla and Wood [16] showed that individuals with higher SC experienced fewer and less severe daily stressful events, compared to individuals who were lower in SC. More recently, using a day reconstruction method, Daly and colleagues [17] demonstrated that self-control was important in the maintenance of stable emotional patterns and it was predictive of low resting heart rate, high heart rate variability and a steep cortisol slope. Therefore, taking these findings together, an aim of the current study was to investigate whether individual differences in trait SC moderated reactivity to a laboratory stressor (described later).

### Primary and Secondary Appraisals of Stress

As outlined above, relatively few studies have explored the relationship between C and stress, and even fewer have investigated how C is associated with *appraisals* of stress (as distinct from the frequency of stressors). An exception is a study by Gartland et al. [18] which showed, for the first time, that the appraisals of daily stressors are influenced by C. Appraisals are the interpretations of events in terms of their benefit or harm for the individual; the transactional model of stress posits two dimensions: primary and secondary appraisals [19]. Primary appraisal involves the evaluation of the risks, demands or challenges of a situation, whilst secondary appraisal evaluates the availability of perceived resources (to cope with the demand) and whether anything can be done to alter the outcome of the situation. In the Gartland et al. [18] study, different facets of C were found to be associated with primary and secondary appraisals of daily stressors, such that *higher* scores on order and industriousness were associated with reporting *more* demanding stressors (higher primary appraisal) and higher responsibility with reporting more perceived resources (higher secondary appraisal). Taken together, these findings suggest that individuals high in C place a *greater* stake in stressors once encountered *and* they have also *greater* confidence in their ability to deal with stressors. This is a noteworthy finding as it suggests that the relationship between C and the appraisal of a stressor may be different from the relationship between C and the number of stressors an individual encounters (e.g. the relationship may be positive in the former case and negative in the latter case). Moreover, in terms of the C and stress appraisal relationship, it might be that individuals high in C, who are highly goal-directed and achievement oriented, have an ‘enhanced salience’ to the identification of stressful encounters (irrespective of the number of stressors they experience), because they perceive stressors in their environment as potential impediments to successfully achieving their goals. As a result, they may be more likely to scan their environment and to identify a potentially stressful episode quickly and perceive it as threatening and demanding, and this helps ensure it is dealt with rapidly and effectively (to protect their goal pursuit). In contrast, it may be that individuals who are low in C are less prone to scan their environment for potential impediments to goal pursuit and as such they are less likely to appraise stressors in their environment as threatening or demanding (because they are less goal-directed and achievement oriented). The current research aimed to further explore these different possibilities.

Finally, a laboratory based stressor was also utilised in this study as this allowed us to explore the relationship between SC, stress appraisals and an objective marker of health (i.e. blood pressure levels). In particular, we were interested in investigating the effects of SC on blood pressure reactivity and recovery from stress. The ‘cardiovascular reactivity hypothesis’ suggests that individuals who have larger blood pressure responses to stress may be more likely to develop health problems such as high blood pressure and cardiovascular disease [20]. For example, in the Coronary Artery Risk Development in Young Adults (CARDIA) study, Matthews and colleagues [21] found that greater cardiovascular reactivity to stress at the beginning of the study was associated with higher blood pressure levels 13 years later. In addition, more recently, researchers have turned their attention to stress recovery because emerging data are suggesting that people who take longer to recover from stress may also be vulnerable to future ill-health (e.g. [22, 23]). In a study by Steptoe and Marmot [23] in which the effects of stress reactivity and post-stress recovery on blood pressure 3 years later were investigated, they showed that increases in blood pressure levels 3 years later were most strongly associated with longer post-stress recovery. More impressively, these effects were independent of all the other risk factors measured (e.g. age, gender, body mass index, socio-economic status, smoking status, hypertension medication).

Therefore, to this end, we used carefully controlled laboratory conditions, to examine the effects of the SC facet on a new stress induction protocol, known as the Maastricht Acute Stress Test (MAST). The MAST was designed to be physiologically and psychologically challenging by combining an *uncontrollable* physical stressor (i.e. a cold pressor challenge) with a social-evaluative (i.e. mental arithmetic) component [24]. We theorised that individual differences in SC are likely to influence responses to the MAST given the uncontrollable nature of the task. More specifically, we predicted that individuals who were high in SC would have better capacity to inhibit the urge to remove their hand from the water bath, be better able to take their time to think about their answers to the mental arithmetic and generally deal better with the physiological and psychological aspects of the MAST.

In summary, this study aimed to build upon and extend the work of Gartland et al. [18] and to examine whether primary and secondary appraisals in relation to a laboratory-based stressor were differentially associated with blood pressure reactivity and recovery in high SC compared to low SC participants. Specifically, we tested whether SC moderated the effects of primary and secondary appraisals on blood pressure responses to the MAST.

## Methods

### Design and Participants

This study employed an adult sample, recruited via emails sent to University of Leeds staff, posters and flyers distributed across the university campus, advertisements on social media and in person. The inclusion criteria for this study were that participants were 18 years or older, spoke fluent English, were generally in good health and not currently taking blood pressure or pain relieving medications. Initially, 679 participants completed the Chernyshenko Conscientiousness Scales (CCS; Green et al. [25]; see below) as a screening measure, with participants indicating the highest and lowest levels of conscientiousness invited to participate in the laboratory study. We aimed to recruit a sample of around 100 participants to the laboratory study based on an earlier study by Gartland, O’Connor and Lawton [18] that explored the relationship between stress appraisals and conscientiousness. We used cut-offs in the current study because we wanted to increase the likelihood of recruiting individuals who reflected ‘true’ high and low C, particularly given that individuals low in C are a difficult group to access. Low conscientiousness was classified as scores equal to 163.2 or below, and high conscientiousness was classified as scores equal to 190.2 or above, based on top and bottom 25% of the sample. Once entered into the laboratory component of the study, scores on the self-control (SC) scale were used to classify participants. In the high SC group, scores ranged from 30 to 38, and in the low SC group, scores ranged from 11 to 27. This double selection was conducted because participants were initially recruited to a larger study exploring the relationship between conscientiousness and health behaviours. In this larger study, there was no strong rationale to recruit on the basis of any one lower order facet. However, as outlined in the ‘Introduction’ section, we felt that there was a strong rationale for selecting participants who were high and low on SC for the current experimental study. One hundred and one participants completed the laboratory study. However, 11 participants were excluded from the analyses due to technical reasons (e.g. not having a baseline blood pressure reading) or because their blood pressure reading was within the hypertensive range (e.g. >140/90 mmHg). The final sample consisted of 46 participants in the low SC group and 44 participants in the high SC group (75 women, mean age of 25.9 years [range = 18–55 years], body mass index mean = 22.25). The sample was largely white ethnicity (78.7%) with the remaining ethnicities including Chinese, Indian, Pakistani, Afro-Caribbean and mixed ethnicities. The study received ethical approval from the School of Psychology’s Ethics Committee (Ref: 14-0016). Participants were compensated with a £15 Love2shop voucher for their time.

### Questionnaire Measures

The CCS (Green et al. [25]) consist of a 60-item questionnaire designed to measure one of the six facets of conscientiousness described by Roberts et al. [14]. The SC subscale consists of 10 items that relate to the propensity to be cautious and able to delay gratification, rather than being impulsive and careless (example items: ‘I rarely jump into something without first thinking about it’, ‘I often rush into action without thinking about potential consequences’, ‘I get into trouble because I act on impulses rather than on thoughts’). The other five facets are industriousness, order, responsibility, traditionalism and virtue. Each item was scored on four-point Likert scale, ranging from 1 (disagree strongly) to 4 (agree strongly). In the current study, participants were initially screened using the total summed score across all six scales (as described above) and mean scores on the SC scale were used to classify participants in the laboratory component. Cronbach’s alphas for the total CCS and the SC subscale were 0.94 and 0.85, respectively.

Stress appraisals were measured via a modified version of the Stressor Appraisal Scale [18, 26]. This scale was delivered in anticipation of the MAST, but after participants viewed a PowerPoint presentation, adapted from Smeets et al. [24], that explained what the upcoming task would involve. Specifically, to begin with, participants were asked to read all of the instructions carefully and to press the space bar to move on to the next page of the PowerPoint presentation. Next, participants were told that the total duration of the task was approximately 12 min, that the water bath beside them contained ice cold water, that during the task they would be asked to place their hand including the wrist joint into the water several times and that in between these ‘trials’ they would have to perform some mental arithmetic. Some of the stress appraisal items were modified to include the word ‘task’ as appropriate. Participants indicated how threatening they thought the ‘challenging task’ was going to be (primary appraisals) and how well they thought they would cope with the task (secondary appraisals). Items were scored on a seven-point Likert scale with responses ranging from ‘not at all’ to ‘to a very large extent’. The primary appraisal items included (1) ‘How threatening do you think the task will be?’, (2) ‘How demanding do you think the task will be?’, (3)‘How stressful do you think the task will be? (4) ‘To what extent do you think you will need to exert yourself to deal with the stress?’ and (5) ‘How much effort (mental or physical) do you think the situation will require you to expend?’ (five items; Cronbach’s *α* = 0.86). The secondary appraisal items included (1) ‘How well do you think you can manage the demands imposed on you by the task?’, (2) ‘How able do you think you are to cope with the task?’ and (3) ‘How well do you think you will perform on the task?’ (three items; Cronbach’s *α* = 0.84). The total score for each scale was calculated to provide an overall primary appraisal score and an overall secondary appraisal score [27].

#### Physical Activity

A number of previous studies have shown that physical activity levels can influence perceived stress ratings and cardiovascular stress reactivity, and they have been found to be associated with conscientiousness [28–30]. Therefore, we included a measure of physical activity in order to control for this variable in our analyses. Specifically, physical activity was assessed in terms of strenuous activity, moderate activity and mild activity. Items were adapted from the International Physical Activity Questionnaire ([31] www.ipaq.ki.se). The following item was initially delivered ‘During a typical 7-day period (a week), how many times on average do you do the following kinds of exercise?’ followed by ‘Strenuous exercise (heart beats rapidly), e.g. running, jogging, hockey, football, squash, basketball, judo, roller skating, vigorous swimming, vigorous long distance bicycling’ and ‘Moderate exercise (not exhausting), e.g. fast walking, baseball, tennis, easy bicycling, volleyball, badminton, easy swimming’. Participants were then required to respond to ‘Number of times per week’ and ‘How much time do you usually spend doing these activities on one of those days (hours/minutes)?’ to each item. The total number of minutes spent undertaking each type of activity per week was then calculated.

### Maastricht Acute Stress Test

The MAST is a recently developed stress protocol designed to be physiologically and psychologically challenging by combining an uncontrollable physical stressor (i.e. a cold pressor challenge) with a social-evaluative (i.e. mental arithmetic) component [24]. A water bath, electrical immersion cooler and circulation pump (lab companion refrigerated bath circulator—JEIO tech model RW-0525G) are used to contain the water and to keep the water at a constant temperature of 2.0 °C. The MAST has been shown to yield similar subjective and cardiovascular stress responses to the Trier Social Stress Test; however, it does not require the presence of a panel (see Kirschbaum et al. [32]). Further details are provided below in the ‘Procedure’ section.

### Blood Pressure Responses to Stress

Systolic blood pressure (SBP) and diastolic blood pressure (DBP) were measured using an Omron M7 upper-arm blood pressure monitor. This device has been clinically validated in terms of reliability and accuracy by major organisations such as the British Hypertension Society (Omron, 2015). SBP and DBP were assessed at baseline (which began after a 10-min rest period), immediately post-MAST procedure and then 10-min post-MAST procedure. At baseline, blood pressure was measured three times. The first measure was discarded (to allow for blood pressure rising in anticipation of having blood pressure measured) and an average of measures two and three were calculated to provide a baseline score. For both the post-MAST and 10-min post-MAST measures, blood pressure was measured twice, with an average of these scores calculated to provide a post-MAST and post-relaxation score. Next, SBP and DBP reactivity and recovery scores were computed for each participant. Blood pressure reactivity was calculated by subtracting the baseline score from the post-MAST score, and blood pressure recovery was calculated by subtracting the post-relaxation score from the baseline score [33].

### Procedure

After completing an online screening questionnaire comprising a demographic questionnaire and the Chernyshenko Conscientiousness Scales (CCS; Green et al. [25]), participants scoring in the top and bottom 25% of the sample for conscientiousness were invited to visit the laboratory. Upon arrival participants were informed that they would be required to complete a challenging task that would last for no longer than 12 min. Participants were also informed that there was a chance that this study may cause some physical discomfort and may cause them to feel stressed. Prior to partaking, participants were informed that they should refrain from consuming alcohol, exercising excessively, or taking any pain medication (e.g. paracetamol, ibuprofen) on the day of testing. Alongside this, participants were asked to re-arrange their appointment if they were feeling unwell (e.g. any cold or flu symptoms).

Once arrived at the laboratory, participants provided informed consent and were allowed to relax for 10 min before having their blood pressure measured (baseline). Participants were then asked to move to a different testing cubicle which housed the cold pressor equipment and a computer for the preparation period. They were told that the total duration of the task was approximately 12 min, that the water bath beside them contained ice cold water, that during the task they would be asked to place their hand including the wrist joint into the water several times and that in between these trials they would have to perform some mental arithmetic. Participants were videotaped throughout and informed the recordings will be used to analyse facial expressions and to compare performance to other participants. Finally, participants were told that the computer would randomly decide the length of time of the trials (i.e. placing hand in water cooler vs performing mental arithmetic); the timing of the trials was fixed and identical for each participant. Following the fifth hand immersion trial, participants were informed that the task was now complete. Participants’ SBP and DBP were then measured immediately post-MAST and again for 10 min (recovery).

### Statistical Analysis

Descriptive statistics and Pearson’s product moment correlation coefficients were computed in order to examine the relationships between the study variables. Multivariate analysis of covariance (MANCOVA) was utilised to examine the main effects of SC group (high SC vs low SC) on BP reactivity to and recovery from stress. For the reasons outlined earlier, physical activity levels were also controlled for in the analyses, although there was no significant difference in mean levels between the high and low C groups (mean minutes per week for strenuous exercise = 79.46, mean minutes per week for moderate exercise = 168.64). Age was also entered as a covariate given that participants in the high SC group were older (*t* (88) = 2.93, *p* = .004) than participants in the low SC group (23.22 vs 28.84 years). Note that BMI was similar across the high and low SC groups and, therefore, not included in any subsequent analyses. Hierarchical linear regression was utilised to test the main moderation hypotheses following the procedures outlined by Kenny et al. [34] using the continuous scores for SC given the evidence highlighting that categorising personality variables that are likely to be dimensional can lead to spurious observations (cf. Ferguson et al. [35]). For each outcome variable (BP reactivity or BP recovery), age, strenuous and mild exercise were entered at step 1, followed by SC score (high vs low) at step 2, stress appraisal variable (primary or secondary appraisal) at step 3 and finally the SC score by stress appraisal multiplicative interaction term entered in step 4. For the regression analyses, all continuous predictor variables were mean centred before entering into the analyses. All analyses were performed in SPSS version 21.0.

## Results

### Descriptive Statistics

Descriptive statistics were calculated for each of the study variables and are presented in Table 1. Inspection of the means indicated that as expected mean scores for SC were significantly higher in the high SC group compared to the low SC group. Primary and secondary appraisal scores, blood pressure levels and reactivity and recovery from the MAST were similar in the high and low SC groups.

**Table 1:** Descriptive statistics for main study variables

	Low SC (*n* = 46)		High SC (*n* = 44)		Total (*n* = 90)		
	Mean	SD	Mean	SD	Mean	SD	*t*
Self-control	24.67	3.58	33.57	2.52	29.02	5.43	13.75**
Primary appraisal	4.62	0.99	4.33	1.14	4.48	1.07	1.29
Secondary appraisal	4.01	1.05	3.99	1.29	4.00	1.16	0.06
Systolic T1	101.37	9.80	103.18	11.78	102.26	10.79	0.80
Systolic T2	109.02	12.27	111.64	17.78	110.30	15.19	0.92
Systolic T3	101.78	10.74	104.06	12.72	102.89	11.74	0.82
Diastolic T1	64.87	7.71	67.78	10.60	66.29	9.29	1.49
Diastolic T2	71.81	10.42	75.74	12.71	73.73	11.70	1.60
Diastolic T3	67.48	8.99	70.99	9.85	69.19	9.53	1.77
Systolic reactivity	7.65	10.24	8.45	12.83	8.04	11.52	0.33
Systolic recovery	0.41	8.08	0.88	8.26	0.64	8.13	0.27
Diastolic reactivity	6.95	9.18	7.97	11.24	7.44	10.20	0.47
Diastolic recovery	2.61	6.96	3.21	7.79	2.91	7.34	0.39

*Note:* T1 = baseline, T2 = post-MAST, T3 = 10-min post-MAST

**p < .001

### Effects of SC Group on Blood Pressure Reactivity and Recovery

MANCOVA confirmed the descriptive statistics and revealed no significant main effects of SC group on SBP or DBP reactivity or recovery from stress (*F*(4, 79) = 0.08, *p* = .99), indicating that at a group level, high and low SC individuals exhibited similar levels of BP reactivity and recovery in response to the MAST.

### The Association Between Stress Appraisals and Blood Pressure Reactivity and Recovery in High SC Versus Low SC Groups

Pearson’s product moment correlations were performed separately in the high SC and low SC groups (see Table 2). As expected, stress appraisals were found to be more strongly associated with BP reactivity and recovery in the high SC group compared to the low SC group. Specifically, primary appraisals were significantly negatively associated with SBP reactivity (*r* = −0.48, *p* = 0.001), SBP recovery (*r* = −0.37, *p* = 0.013) and with DBP reactivity (*r* = −0.37, *p* = 0.013) in the high SC group, but not in the low C group. Secondary appraisals were not significantly associated with BP reactivity or recovery in either group.

**Table 2:** Pearson’s correlations between stress appraisals and blood pressure reactivity and recovery in high self-control and low self-control groups

	High self-control group		Low self-control group	
	Primary appraisal	Secondary appraisal	Primary appraisal	Secondary appraisal
SBP reactivity	−0.48**	0.16	0.04	−0.21
SBP recovery	−0.37*	0.19	0.09	−0.01
DBP reactivity	−0.37*	0.18	0.07	−0.19
DBP recovery	−0.11	0.00	0.04	−0.12

*SBP* systolic blood pressure, *DBP* diastolic blood pressure

*p < 0.05; **p < 0.01

### Moderating Effects of Self-Control on Stress Appraisal—Blood Pressure Reactivity and Recovery Relations

**Fig. 1:**
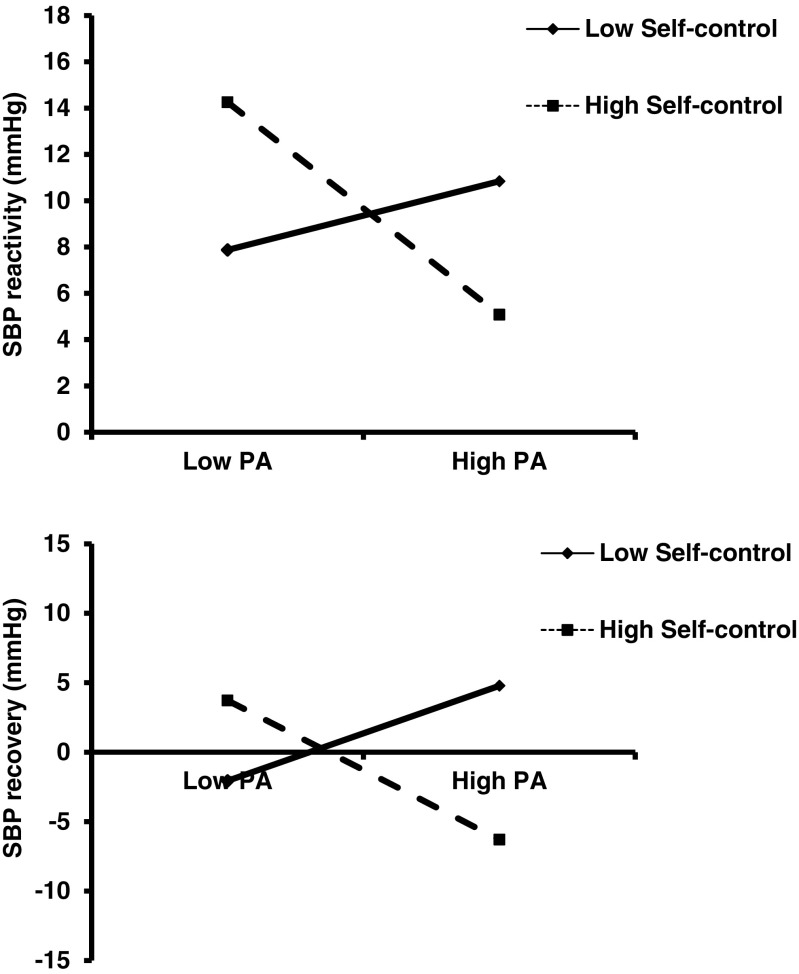
Interactive effects of trait self-control and primary appraisal (*PA*) on systolic blood pressure (*SBP*) reactivity (*upper panel*) and recovery (*lower panel*)

The hierarchical regression analyses were only conducted involving primary appraisals given that secondary appraisals were not associated with BP outcomes in high or low SC groups (see Table 3). For SBP reactivity, at step 1 (age, strenuous and moderate exercise) and step 2 (SC group), none of the variables significantly entered the equation. However, at step 3, primary appraisal significantly explained 6% of the variance (*F*(1, 86) = 5.95, *p* = 0.017), such that higher levels of primary appraisal were associated with lower SBP in response to the MAST. Next, at step 4, when the primary appraisal x SC group interaction term entered the equation, it explained an additional 7% of the variance in SBP reactivity (*F*(1, 85) = 6.50, *p* = 0.013). The two-way interaction is depicted in Fig. 1 following procedures outlined by Aiken and West [36] and Dawson [37]. Inspection of the figure (upper panel) shows that participants in the high SC group who reported higher levels of primary appraisal exhibited lower SBP in response to the MAST. Different levels of primary appraisals were not associated with SBP levels in low SC group. It is important to note that individuals high in C who reported *lower* primary appraisals showed higher SBP reactivity; however, this response reflects the normal stress response observed in reactivity studies (cf. Smeets et al. [17]).

**Table 3:** Hierarchical regression analyses testing the interactive effects of trait self-control (using continuous scores) and primary appraisals on blood pressure reactivity and recovery (*n* = 90)

		*β* step 1	*β* step 2	*β* step 3	*β* step 4	Δ*R* ^2^ for step	Total *R* ^2^
	**SBP reactivity**						
Step 1	Age Strenuous exercise Moderate exercise	0.16 0.08 −0.05	0.18 0.08 −0.05	0.15 0.04 −0.03	0.14 0.07 0.00	0.036	
Step 2	Self-control (SC) score		−0.05	−0.07	−0.05	0.000	
Step 3	Primary appraisal (PA)			−0.25*	−0.20	0.056	
Step 4	SC score × PA				−0.24*	0.056*	0.150
	**SBP recovery**						
Step 1	Age Strenuous exercise Moderate exercise	0.03 0.08 0.03	0.03 0.08 0.03	0.01 0.05 0.04	−0.01 0.06 07	0.007	
Step 2	Self-control (SC) score		0.01	−0.00	0.01	0.010	
Step 3	Primary appraisal (PA)			−0.15	0.11	0.024	
Step 4	SC score × PA				−0.35*	0.091**	0.132
	**DBP reactivity**						
Step 1	Age Strenuous exercise Moderate exercise	0.10 0.14 0.06	0.12 0.14 0.06	0.10 0.11 0.08	0.09 0.14 0.10	0.031	
Step 2	Self-control (SC) score		−0.06	−0.07	−0.05	0.003	
Step 3	Primary appraisal (PA)			−0.17	−0.13	0.027	
Step 4	SC score × PA				−0.22*	0.046*	0.106
	**DBP recovery**						
Step 1	Age Strenuous exercise Moderate exercise	0.01 0.11 −0.01	0.08 0.12 −0.00	0.07 0.12 0.00	0.06 0.13 0.02	0.013	
Step 2	Self-control (SC) score		0.22	−0.22	−0.21	0.041	
Step 3	Primary appraisal (PA)			−0.04	−0.02	0.002	
Step 4	SC score × PA				−0.14	0.018	0.074

*p < .05; **p < .01

For SBP recovery, at step 1 (age, strenuous and moderate exercise), step 2 (SC group) and step 3 (primary appraisal), none of the variables significantly entered the equation. However, at step 4, when the primary appraisal × SC group interaction term entered the equation, it explained an additional 5% of the variance in SBP recovery (*F*(1, 85) = 4.42, *p* = 0.038). The two-way interaction is depicted in Fig. 1 (lower panel) and shows that participants in the high SC group who reported higher levels of primary appraisal had lower SBP recovery values following the MAST (indicative of better recovery levels).

**Fig. 2:**
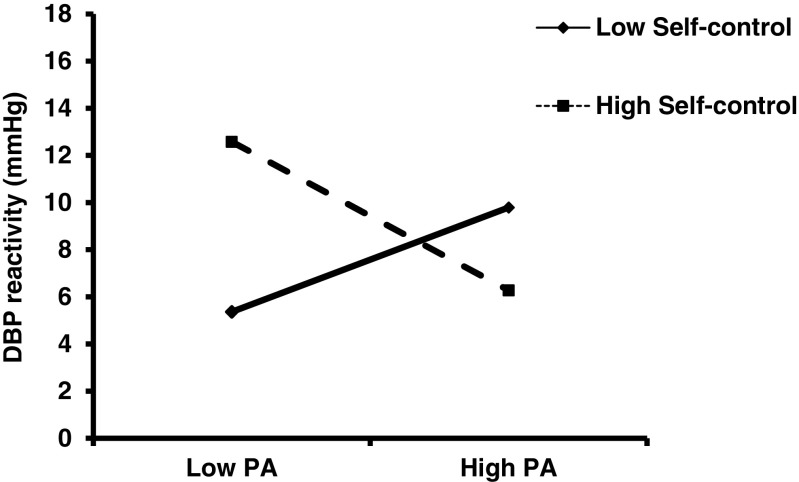
Interactive effects of trait self-control and primary appraisal (*PA*) on diastolic blood pressure *(DBP*) reactivity

For DBP reactivity, at step 1 (age, strenuous and moderate exercise), step 2 (SC group) and step 3 (primary appraisal), none of the variables significantly entered the equation. However, again, at step 4, when the primary appraisal × SC group interaction term entered the equation, it explained an additional 5% of the variance in DBP reactivity (*F*(1, 85) = 4.37, *p* = 0.04). The interaction is depicted in Fig. 2 and shows a similar pattern such that participants in the high SC group who reported higher levels of primary appraisal exhibited lower DBP in response to the MAST.

For DBP recovery, none of the variables significantly entered the equation at any of the steps.

## Discussion

The main findings that emerged from the current study were that SC group significantly interacted with primary appraisals, such that higher primary appraisals were associated with lower SBP and DBP reactivity in the high SC group but not the low SC group. In addition, lower SBP recovery values, indicative of better recovery, were observed in the high SC group compared to their low SC counterparts. These findings may be important because they suggest that SC, a lower-order facet of C, may influence health and well-being by modifying the effects of stress appraisal processes on BP reactivity and recovery from stress.

These results are in line with Gartland et al.’s [18] study, where facets of C were found to be more strongly associated with primary appraisals compared to the secondary appraisals. However, the current results also demonstrate, for the first time, that the effects of higher levels of primary appraisals reported by participants higher in SC may extend to important objective measures of health (i.e. lower BP reactivity to stress). This is a noteworthy observation as it suggests a possible pathway through which one facet of C, namely, SC, may help protect health and promote longevity in the longer term in high C individuals. For example, the ‘cardiovascular reactivity hypothesis’ suggests that individuals who have greater physiological responses to stress are more likely to develop health problems in the future [28, 38]. In particular, individuals who exhibit greater increases in blood pressure (and/or heart rate) after stressful situations may be at greater risk of developing high blood pressure and cardiovascular disease.

The current results are also partially in keeping with our notion of ‘enhanced salience’ to the identification of stressful encounters as a potential mechanism that differentiates high SC individuals from low SC individuals in the context of stress appraisals. We speculated that individuals high in SC would perceive the laboratory stressor as more threatening and demanding because they have a propensity to be highly goal-directed and achievement oriented, and as such, they are likely to be motivated to deal with the stressor quickly and effectively in order to move onto their next goal. As outlined earlier, we did find a significant interaction between SC and primary appraisals in the hypothesised direction; however, we did not find significantly greater levels of primary appraisals in the high SC compared to low SC individuals. Therefore, to our mind, these results highlight that the interactive relationship between SC and primary appraisals is more complex than we initially theorised. An important next step would be to replicate the current findings with other laboratory-based stressors and implicit and explicit measures of appraisals in order to explore precisely how high levels of SC and primary appraisals interact to transmit their positive effects and whether individuals low in SC fail to respond to other types of stressors. Another fruitful line of investigation would be to utilise daily diary and experience sampling techniques to investigate whether SC moderates the effects of different daily stressors on hemodynamic processes [27, 39].

Surprisingly, we also found that SC was not related to secondary appraisals (or blood pressure reactivity or recovery), which are the individual’s perceptions of the availability of the resources they have to cope with the demand. Earlier work by Gartland et al. [18] showed that the lower-order facet of responsibility (but not other facets including SC) was associated with perceived resources in relation to the most stressful event participants encountered in the past week. Other research has indicated that measures of total C are related to the employment of specific coping strategies (which are very different from the secondary appraisal measure incorporated here). Total C was shown to be positively associated with the use of approach style behaviours such as problem solving, cognitive restructuring, emotional social support, instrumental social support and emotion regulation [40–42] whilst negatively associated with avoidant style behaviours such as denial, negative emotion-focused, avoidant coping and substance use as forms of coping [40]. Furthermore, research from O’Brien and DeLongis [s] demonstrated that individuals who were high in total C employed less escape-avoidance and self-blaming strategies, when assessed over a range of situations. These authors also suggested that differences in coping style and coping strategies may be due to the way in which stressful situations are appraised [43]. Nevertheless, the absence of coping relations in the current study may be accounted for by the nature of the secondary appraisals measure used in this study and/or the utilisation of an uncontrollable, laboratory stressor. For example, participants may have found it difficult to rate in advance the extent to which they think they could manage the demands imposed on them by the task (although, they did view a PowerPoint presentation beforehand outlining the precise nature of the stress task). Indeed, the mean scores on the secondary appraisals subscale were markedly lower in the current study compared to the earlier Gartland et al. [18] study (4.81 vs 4.00). In addition, it is worth noting that the majority of previous research into personality and coping relations has incorporated measures of coping strategies and styles and not of secondary appraisal specifically, thereby, making comparisons with the broader coping literature difficult (cf. [40]). Therefore, it would be helpful if future research gave additional consideration to how secondary appraisals are assessed in the context of laboratory-based stress induction paradigms as well as establishing how they relate to conventional measures of coping strategies and styles.

We recognise that there are a number of shortcomings and limitations of the current study that require further comment. We note that the sample size was relatively small and that additional effects may have been observed in a larger study. We are also mindful that participants in the high SC group were significantly older than their low SC counterparts. As a result, we controlled for age in all analyses; however, it is difficult to theorise how the high SC group being older might account for the observed effects. Nevertheless, future research ought to attempt to match for age. It is worth noting that despite selecting participants from the upper and lower quartiles for SC, it is likely that participants in the low SC group did not represent truly very low SC participants as the mean score was not at the very bottom of the range for possible SC scores. Such participants are difficult to engage given that they are unlikely to be interested in participating in scientific studies. Nevertheless, future research ought to endeavour to identify, target and recruit participants at this end of the distribution. We acknowledge also that given we did not utilise a minute-to-minute measure of BP recovery, we are unable to say anything about the fine-grained dynamics of recovery (e.g. in relation to the speed of blood pressure recovery following the stressor). Finally, we recognise that before firm conclusions can be drawn and to ensure the current findings are not spurious, they need to be replicated using different stressors, additional measures of stress appraisals and further objective and subjective outcome measures.

In summary, the results of the current study showed that SC group significantly interacted with primary appraisals, such that higher primary appraisals were associated with lower SBP and DBP reactivity and lower SBP recovery values in the high SC group compared to the low SC group. These findings may be important because they suggest that SC may influence health and well-being by modifying the effects of stress appraisal processes on BP reactivity and recovery from stress.

## References

[1] Friedman HS, Tucker JS, Tomlinson-Keasey C, Schwartz JE, Wingard DL, Criqui MH (1993). Does childhood personality predict longevity?. J Pers Soc Psychol.

[2] Bogg T, Roberts BW (2004). Conscientiousness and health-related behaviors: a meta-analysis of the leading behavioral contributors to mortality. Psychol Bull.

[3] Kern ML, Friedman HS (2008). Do conscientious individualss live longer? A quantitative review. Health Psychol.

[4] Molloy GJ, O’Carroll RE, Ferguson E (2014). Conscientiousness and medication adherence: a meta-analysis. Ann Behav Med.

[5] Friedman HS, Tucker JS, Schwartz JE, Martin LR, Tomlinson-Keasey C, Wingard DL (1995). Childhood conscientiousness and longevity: health behaviors and cause of death. J Pers Soc Psychol.

[6] Goodwin RD, Friedman HS (2006). Health status and the five-factor personality traits in a nationally representative sample. J Health Psychol.

[7] Jokela M, Hintsanen M, Hakulinen C, Batty GD, Nabi H, Singh-Manoux A, Kivimaki M (2013). Association of personality with the development and persistence of obesity: a meta-analysis based on individual-participant data. Obesity Rev.

[8] Ferguson E (2013). Personality is of central concern to understand health: towards a theoretical model for health psychology. Health Psychol Rev.

[9] Gartland N, O’Connor DB, Lawton R, Ferguson E (2014). Investigating the effects of conscientiousness on daily stress, affect and physical symptom processes: a daily diary study. Br J Health Psychol.

[10] Hagger-Johnson G, Berwick B, Conner M, O’Connor DB, Shickle D (2012). School-related conscientiousness, alcohol drinking and cigarette smoking in a representative sample of English school pupils. Br J Health Psychol.

[11] Hagger-Johnson GE, Sabia S, Nabi H, Brunner E, Kivimaki M, Shipley M (2012). Low conscientiousness and risk of all-cause, cardiovascular and cancer mortality over 17 years: Whitehall II cohort study. J Psychosom Res.

[12] Luo J, Roberts BW (2015). Concurrent and longitudinal relations among conscientiousness, stress, and self-perceived physical health. J Res Pers.

[13] O’Connor DB, Conner M, Jones F, McMillan B, Ferguson E (2009). Exploring the benefits of conscientiousness: an investigation of the role of daily stressors and health behaviors. Ann Behav Med.

[14] Roberts BW, Chernyshenko OS, Stark S, Goldberg LR (2005). The structure of conscientiousness: an empirical investigation based on seven major personality questionnaires. Personnel Psychol.

[15] Hofmann W, Friese M, Strack F (2009). Impulse and self-control from a dual-systems perspective. Per Psycho Sci.

[16] Galla BM, Wood JJ (2015). Trait self-control predicts adolescents’ exposure and reactivity to daily stressful events. J Pers.

[17] Daly M, Baumeister RF, Delaney L, MacLachlan M (2014). Self-control and its relation to emotions and psychobiology: evidence from a day reconstruction method study. J Behav Med.

[18] Gartland N, O’Connor DB, Lawton R (2012). The effects of conscientiousness on the appraisals of daily stressors. Stress Health.

[19] Lazarus RS, Folkman S (1984). Stress, appraisal, and coping.

[20] Kamarck TW, Lovallo WR (2003). Cardiovascular reactivity to psychological challenge: conceptual and measurement considerations. Psychosom Med.

[21] Matthews KA, Katholi CR, McCreath H, Whooley MA, Williams DR, Zhu S, Markovitz JH (2004). Blood pressure reactivity to psychological stress predicts hypertension in the CARDIA study. Circulation.

[22] Schneider GM, Jacobs DW, Gervitz RN, O’Connor DT (2003). Cardiovascular haemodynamic response to repeated mental stress in normotensive subjects at genetic risk of hypertension: evidence of enhanced reactivity, blunted adaption and delayed recovery. J Hum Hyperten.

[23] Steptoe A, Marmot M (2005). Impaired cardiovascular recovery following stress predicts 3-year increases in blood pressure. J Hyperten.

[24] Smeets T, Cornelisse S, Quaedflieg C, Meyer T, Jelicic M, Merckelbach H (2012). Introducing the Maastricht Acute Stress Test (MAST): a quick and non-invasive approach to elicit robust autonomic and glucocorticoid stress responses. Psychoneuroendocrinology.

[25] Green J, O’Connor DB, Gartland N, Roberts BW (2016). The Chernyshenko conscientiousness scales: a new facet measure of conscientiousness. Assessment.

[26] Schneider TR (2008). Evaluations of stressful transactions: what’s in an appraisal?. Stress Health.

[27] Gartland N, O’Connor DB, Lawton R, Ferguson E (2014). Investigating the effects of conscientiousness on daily stress, affect and physical symptom processes: a daily diary study. Br Health Psychol.

[28] Forcier K, Stroud L, Papandonatos G, Hitsman B, Reiches M, Krishnamoorthy J, Niaura R (2006). Links between physical fitness and cardiovascular reactivity and recovery to psychological stressors: a meta-analysis. Health Psych.

[29] Lutz RS, Lochbaum MR, Lanning B (2007). Cross-lagged relationships among leisure-time exercise and perceived stress in blue-collar workers. J Sport Exer Psychol.

[30] Sutin AR, Stephan Y, Luchetti M, Artese A, Oshio A, Terracciano A (2016). The five-factor model of personality and physical inactivity: a meta-analysis of 16 samples. J Res Pers.

[31] Craig CL (2003). International physical activity questionnaire: 12-country reliability and validity. Med Sci Sports Exerc.

[32] Kirschbaum C, Pirke KM, Hellhammer DH (1993). The ‘trier social stress test’—a tool for investigating psychobiological stress responses in a laboratory setting. Neuropsychobiology.

[33] Menkes MS, Matthews KA, Krantz DS, Lundberg U, Mead LA, Qaqish B, Pearson TA (1989). Cardiovascular reactivity to the cold pressor test as a predictor of hypertension. Hypertension.

[34] Kenny DA, Kashy DA, Bolger N, Gilbert DT, Fiske ST, Lindzey G (1998). Data analysis in social psychology. The handbook of social psychology.

[35] Ferguson E, Williams L, O’Connor RC, Howard S, Hughes B, Johnston DW, Hay J, O’Connor DB, Lewis CA, Grealy MA, O’Carroll RE (2009). A taxometric analysis of type-D personality. Psychosom Med.

[36] Aiken LS, West SG (1991). Multiple regression: testing and interpreting interactions.

[37] Dawson JF (2014). Moderation in management research: what, why, when and how. J Bus Psychol.

[38] Abraham C, Conner M, Jones F, O’Connor DB (2016). Health Psychology: topics in applied psychology.

[39] Gartland N, O’Connor DB, Lawton R, Bristow M (2014). Exploring day-to-day dynamics of daily stressor appraisals, physical symptoms and the cortisol awakening response. Psychoneuroendocrinology.

[40] Connor-Smith JK, Flachsbart C (2007). Relations between personality and coping: a meta-analysis. J Pers Soc Psychol.

[41] Vollrath M, Torgersen S (2000). Personality types and coping. Pers Indiv Diff.

[42] Bartley CE, Roesch SC (2011). Coping with daily stress: the role of conscientiousness. Pers Indiv Diff.

[43] O’Brien TB, DeLongis A (1996). The interactional context of problem-, emotion- and relationship-focused coping: the role of the big five personality factors. J Pers.

